# Treatment of breast cancer patients from a public healthcare system in a private center: costs of care for a pilot public-private partnership in oncology

**DOI:** 10.1590/S1679-45082013000200014

**Published:** 2013

**Authors:** Rafael Aliosha Kaliks, Lucíola de Barros Pontes, Cinthia Leite Frizzera Borges Bognar, Kelly Cristine Carvalho Santos, Sílvio Eduardo Bromberg, Paulo Gustavo Tenório do Amaral, Theodora Karnakis, Michael Chen, Cláudia Toledo de Andrade, Joacira Dantas, Daísa de Mesquita Escobosa, Auro Del Giglio

**Affiliations:** 1 Hospital Israelita Albert Einstein, São Paulo, SP, Brazil

**Keywords:** Breast neoplasms/economy, Public-private partnership, Healthcare costs

## Abstract

**Objective:**

To describe the flow and costs associated with the diagnosis and treatment of patients with breast cancer who come from the public healthcare system and were treated at *Hospital Israelita Albert Einstein*.

**Methods:**

Between August 2009, and December 2011, 51 patients referred by the Unified Public Healthcare System (SUS) had access to *Hospital Israelita Albert Einstein* for diagnostic radiology, medical oncology, radiotherapy, and oncologic/ breast reconstruction surgery. The data were collected retrospectively from the hospital records, patient charts, pharmacy records, and from the hospital billing system.

**Results:**

The total sum spent for diagnosis and treatment of these 51 patients was US$ 1,457,500.00. This value encompassed expenses with a total of 85 hospitalizations, 2,875 outpatient visits, 16 emergency room visits, and all expenses associated with these stays at the hospital. The expenditure for treatment of each patient submitted to biopsy, breast conserving surgery, adjuvant chemotherapy without trastuzumab (a regime with taxane followed by anthracycline), radiotherapy, and 5 years of tamoxifen was approximately US$ 25,500.00.

**Conclusion:**

Strategies for cost-reduction of treatment in the private setting are necessary to enable future large-scale public-private partnerships in oncology.

## RESUMO

**Objetivo:**

Descrever os fluxos e os custos associados ao diagnóstico e tratamento de pacientes com câncer de mama provenientes do Sistema Único de Saúde e tratadas no Hospital Israelita Albert Einstein.

**Métodos:**

Entre agosto de 2009 e dezembro de 2011, 51 pacientes do Sistema Único de Saúde tiveram acesso à estrutura do Hospital Israelita Albert Einstein para os serviços de radiologia, oncologia, radioterapia e cirurgia oncológica/reconstrutora de mama. Os dados foram coletados retrospectivamente, a partir do registro hospitalar de câncer, prontuários, registros de farmácia e do sistema de cobrança do hospital.

**Resultados:**

O valor total gasto para o diagnóstico e tratamento dessas 51 pacientes foi de R$ 2.915.000,00. Esse valor compreendeu os gastos com um total de 85 internações, 2.875 atendimentos ambulatoriais, 16 atendimentos no pronto atendimento e todos os custos associados a essas passagens pelo hospital. O valor gasto para o tratamento de cada paciente submetida à biópsia, setorectomia, quimioterapia adjuvante sem trastuzumabe (esquema com taxano seguido de antraciclina), radioterapia e 5 anos de tamoxifeno, foi de, aproximadamente, R$ 51.000,00

**Conclusão:**

Estratégias de redução do custo do tratamento na rede privada seriam necessárias para permitir futuras parcerias público-privadas em grande escala, em oncologia.

## INTRODUCTION

In Brazil, according to the estimates of the National Cancer Institute *[Instituto Nacional de Câncer]* (INCA), during the year 2012, approximately 518,510 new cases of cancer were diagnosed^(1)^. In the female Brazilian population, excluding non-melanoma skin cancer, breast cancer is the neoplasm with the highest incidence (52/100,000women) and the primary cause of cancer-related mortality^(1)^. On the other hand, the Unified Healthcare System *[Sistema* Único *de Saúde]* (SUS) has too limited a structure to be able to adequately diagnose and treat all these new cases^(2)^, thus generating deficiencies in tracking and diagnosis, with consequent delay at various phases of treatment, leading to a negative impact on the prognosis of patients with cancer^(3)^. Published data have demonstrated that in public health system there are delays between the first symptoms of the disease and the establishment of the diagnosis, between the diagnosis and treatment, and among the various phases of treatment^(3,4)^. These delays can lead to worse outcomes, including in lower survival rates^(5,6)^. Within this context, the structural deficiency of SUS could be partially compensated by the establishment of public-private partnerships^(6,7)^(PPP) between governments and private hospitals, which could absorb part of the cases for immediate treatment until the public service is ready to care for all the demands with no delays. In order for a partnership such as this to be possible, it is necessary to know the details of the costs of breast cancer treatment.

With the aim of better assisting SUS and establishing the actual costs of such a PPP, in 2009, *Hospital Israelita Albert Einstein* (HIAE) initiated the Program for Oncologic Patients (PRPO). The program assisted SUS in the training of human resources related to the oncology screening, diagnosis and treatment, in addition to making available the HIAE structure for the diagnosis and treatment of breast cancer patients. These patients used HIAE from the diagnosis through the surgical intervention, radiotherapy and chemotherapy, until follow-up once treatment was ended. The program also had the objective of promoting research by investigating the costs of breast cancer treatment, following the best international standards(^8^), in order to collect this information to be used as a basis for future PPPs in this area.

## OBJECTIVE

To describe patient flow between SUS and HIAE and to evaluate the costs associated with diagnosis and treatment of patients with breast cancer seen by the PRPO, in order to allow planning of future social projects of public-private partnerships.

## METHODS

Initially, women with radiological alterations of the breast (BIRADS≥4) coming from the Basic Healthcare Unit (UBS) in *Vila das Belezas* and from the Specialties Outpatient Clinic in Pirajussara, both located in the Southern area of the city of São Paulo (SP), were referred to HIAE for breast biopsies, and then again referred to the public network after the procedures. Once the diagnosis of breast cancer was confirmed, patients selected as per the judgment of the original UBS were directed to the Oncology and Hematology Center of HIAE for treatment. For a 30-month period, the PRPO offered the structure of HIAE so that these women could have access to the services of radiology, oncology, radiotherapy, oncologic surgery, and breast reconstruction, besides supplementary tests (breast biopsy, mammogram, ultrassound, laboratory and other imaging tests deemed necessary).

Once integrated into the program, the patients followed the flow described below.

### Initial evaluation

At the first visit, all the patients were evaluated by a breast surgeonnece and a nurse, who determined the necessary tests and the subsequent. Subsequently, all the cases discussed at a multidisciplinary meeting between medical oncologists, breast surgeons, pathologist, radiologists, radiotherapists, nurses, dieticians, psychologists, nursing students, and oncology medical residents. This weekly multidisciplinary discussion occurred before treatment in all cases except those in which the chemotherapeutical treatment needed to be started urgently, even before the weekly meeting. The discussions of cases by the multidisciplinary team allowed the establishment of an institutionally standardized plan, with a optimal chance of success. Standard staging consisted of an abdominal ultrasound and chest X-ray. In specific cases of patients with clinical complaint, or for those with tumors >5cm, a clinically axillary involvement or after confirmation of surgery of more than four compromised lymph nodes, staging was complemented by computed tomography of the abdomen and chest (or positron emission tomography – PET-CT – in special situations), and bone scan.

### Surgical treatment

All the patients with surgical indication were operated on after the multidisciplinary discussion. Breast conserving surgery was recommended for all the initial cases (small lesions that allowed the removal of the tumor surrounded by a margin of healthy tissue) and for those who presented with a response to neo-adjuvant therapy, with a resulting lesion that allowed the removal of the tumor surrounded by a margin of healthy tissue^(8)^. All the patients underwent sentinel lymph node biopsy (SLN), with axillary lymph node dissection in case there was presence of macrometastases in the SLN. In patients who received neo-adjuvant therapy, the SLN biopsy was performed at the time ofsurgery. For patients submitted to mastectomy, the surgical plan included immediate reconstruction, with planning made together by the breast surgeon and a plastic surgeon. Selected cases (young patients, suspected multifocal disease, or yet with suspected bilateral cancer) were submitted to magnetic resonance imaging of the breast for surgical planning.

### Systemic treatment

Systemic treatment followed the protocol recommended internationally by the National Comprehensive Cancer Network (NCCN)^(8)^. The rare cases in which there was a deviation from these guidelines were a result of multidisciplinary discussion, and the consequence of limitations imposed by comorbidities or by characteristics detected in the geriatric assessment of the patients.

### Radiation therapy

Radiation therapy was recommended for all the women submitted to conservative surgery, for those with tumors larger than 5cm and/or with four or more positive lymph nodes in the axilla^(8)^. For women who received neo-adjuvant systemic treatment, the indication for radiotherapy was dictated by the original clinical staging rather thanthe pathological staging.

### Multidisciplinary follow-up

All the patients underwent evaluation and were subsequently followed by psychologist, nutrition specialist, and physical therapy & rehabilitation treatmentas needed. Patients older than 60 years of age were evaluated preoperatively by a geriatrician, and had a geriatric follow up during treatment. In cases with a family history for cancer or when clinical evaluation raised the suspicion of a hereditary syndrome of predisposition to cancer, the patients were evaluated by the Genetic Counseling Service at HIAE. After the end of adjuvant chemotherapy and radiation therapy, all the patients were followed every three months or more frequently, depending on clinical necessity.

### Data collection

Once the patient was enrolled in this programe until the end of treatment, the dates of tests and procedures carried out were tabulated in real-time, allowing the measurement of time intervals between the various phases of care. The team from the Hospital Cancer Registrar, responsible for collecting the data from all the oncologic patients treated at the hospital, tabulated part of the data; the remaining data were obtained retrospectively from patient charts, pharmacy records, and from the billing system of the hospital. All the monetary values are in reference to costs in US Dollars, paid by the PRPO for every procedure. Collection of clinical data was allowed after thefrom the Ethics in Research Committee of HIAE, filed under protocol # CAAE 11902312.7.0000.0071.

## RESULTS

Between August 2009 and December 2011, 96 patients were referred by the public health system for investigation of an abnormal mammogram. All 96 patients were biopsied and then referred with the biopsy result back to the original UBS. Of those 96, 51 were redirected for treatment by PRPO. Table 1 describes the characteristics of these 51 patients at diagnosis. The median age of these patients was 55 years (32 to 82 years).

**Table 1:** Clinical characteristics of 51 patients with breast cancer treated by the Program for Oncologic Patients

Clinical characteristics		n (%)
		51 (100)
Immunohistochemistry*		
	ER+ and/or PR+	43 (84)
	Her2+	14 (27)
	Triple negative	8 (16)
Clinical staging		
	*In situ*	3 (6)
	I-II	31 (61)
	III	13 (25)
	IV	4 (8)
Menopausal status		
	Pre	17 (33)
	Post	34 (67)

* There is overlapping of hormone receptor and Her2 positive tumors.

ER: estrogen receptor; PR: progesterone receptor.

Table 2 shows the number of therapeutic procedures, from the onset of the project until December 2011. Of the 51 patients, only 2 died as a consequence of the disease, and the other 49 remained in treatment or follow-up until December 31, 2011. The median time of follow-up until this date was 22 months.

**Table 2:** Therapeutic procedures performed in patients treated by the Program for Oncologic Patients

Treatment		n (%)
		51 (100)
Initial treatment		
	Mastectomy	13 (25.5)
	Breast conserving surgery	17 (33.3)
	Neo-adjuvant chemotherapy	15 (29.5)
	Neo-adjuvant hormone therapy	2 (3.9)
	Palliative systemic treatment	4 (7.8)
Radiation therapy		
	Adjuvant	31 (60.8)
	Palliative	3 (5.9)
	Not indicated	17 (33.3)
Neo-adjuvant treatment		17 (100)
	Chemotherapy based on anthracycline + taxane	5 (29.4)
	Chemotherapy based on anthracycline + taxane + trastuzumab	9 (53)
	Hormone therapy	2 (11.7)
	Hormone therapy + trastuzumab	1 (5.9)
Adjuvant treatment		n (%)
Chemotherapy		13 (100)
	Based on anthracycline + taxane	7 (53.9)
	Based on anthracycline	1 (7.7)
	Based on taxane	5 (38.4)
	Anti-Her-2	13 (100)
	Trastuzumab during one year	13 (100)
Hormone therapy		35 (100)
	Aromatase inhibitor	17 (50)
	Tamoxifen	18 (50)
Palliative systemic treatment		9* (NA**)
	Hormone therapy	5 (−)
	Chemotherapy	9 (−)
	Trastuzumab	1 (−)

* In the follow-up period, 5 patients developed metastatic disease, totaling up to 9 patients receiving palliative systemic treatment;

** NA: not applicable. The patients on palliative systemic treatment received more than one therapeutic modality, which did not allow analyzing percentage in relation to the total number.

Mastectomies and breast conserving surgeries determined hospitalization with a median duration of 2 days (varying from 1 to 7 days). There was no death or life-threatening adverse event related to the toxicity of the systemic treatment or resulting from complications of the surgical treatment.

Throughout 2009 to 2011, 72 multidisciplinary meetings were held to discuss the cases. During the same period, there were 85 hospitalizations (including those for surgery) and 2,875 visits (a sum of medical visits from any specialty related to treatment, the psychologist and dietician visits). Table 3 shows the cost associated with the items of diagnosis, staging, and treatment. It is important to point out that these are the minimal costs, they do not include fees of the professionals involved. Radiological tests have the costs of depreciation of the equipment accounted for. Magnetic ressonance imaging of the breasts was requested for additional investigation and/or surgical planning in 21 patients. Since few patients presented with metastatic diseases, and the cost of this treatment is extremely variable, we did not discriminate the cost for this portion of patients on table 3, although it is counted in the total sum of expenses described.

**Table 3:** Cost per procedure per breast cancer patient treated by the Program for Oncologic Patients

Cost per procedure		Cost/patient (Us$)*
Diagnosis		
	Breast biopsy (needle + procedure)	503.50
	Pathology with IHC in core Bx	158.50
	Frozen biopsy + pathology with IHC	770.00
	FISH for Her2	506.00
Staging and/or follow-up		
	Chest X-ray + abdominal US	325.50
	Thorax, abdomen and pelvis CT	368.00
	Bone scintigraphy	139.50
	Breast MRI	505.00
Surgical treatment **		
	Mastectomy/quadrantectomy	6,062.50
	Port-a-cath	2,248.00
Radiation therapy		
	Adjuvant	5,040
	Palliative (for pain relief/CNS)	2,840.50
Adjuvant treatment ***		
	Taxane > anthracycline + ciclophosphamide	11,194.00
	Anthracycline + ciclophosphamide > taxane + trastuzumab	82,175.00
	Docetaxel + ciclophosphamide	7,960.00
	Fluorouracil + anthracycline + ciclophosphamide	645.00
	Trastuzumab/year	66,250.00
	Anastrazol/year	3,842.50
	Tamoxifen/year	547.50
Admissions to hospital (mean cost/admission)****		3,850.50

* Exchange rate: R$ 1.00 = US$ 0.50;

** includes operating theatre costs, but not professional fees;

*** price excludes premedication; value calculated for body surface area (BSA) of 1.6m^2^;

**** admissions not scheduled (adverse reactions, infections and clinical and surgical complications).

IHC: immunohistochemistry; BX: biopsy; FISH: *in situ* hybridization; US: ultrasound; CT: computed tomography; MRI: magnetic resonance imaging; CNS: Central Nervous System.

The total ammount spent by PRPO between 2009 and 2011 for the diagnosis and treatment of these 51 patients was US$ 1,457,500.00. Besides the items described on table 3, this value included expenses with a total of 85 hospitalizations, 2,875 outpatient visits, 16 treatments at the emergency department (generating 12 unplanned hospitalizations), costs of the supportive care medications used during chemotherapy (antiemetic and pre-medications, colony-stimulating factors, antibiotics, and bisphosphonates), and all the costs associated with these hospital stays.

Another documented parameter was the evaluation of the intervals between the various phases of diagnosis and treatment, as shown in table 4.

**Table 4:** Intervals between diagnosis and treatment phases for breast cancer patient treated by the Program for Oncologic Patients

Interval	Median (in days)	Min - max (in days)
First abnormality* to diagnosis	166	13-744
Pathological diagnosis to first treatment**	68	11-435
End of neo-adjuvant therapy to surgery	52	12-358
Surgery to beginning of adjuvant therapy	34	14-134
End of systemic treatment to radiation therapy	43	18-136

* First change: patient reported on self-exam, professional examination or altered imaging;

** surgery or chemotherapy. Min.: minimum; max.: maximum.

## DISCUSSION

The establishment of auxiliary programs to SUS through PPP, including those that are mostly clinical, may assist not only in training of staff from the public system, but also may help diminish the number of patients with cancer who await treatment^(7)^. PRPO was set up including one arm dedicated to clinical assistance, in which 51 women with breast cancer were treated at HIAE. Since they were patients coming from a Basic Health Unit Service and from a SUS Specialties Outpatient Clinic, they are a small, but representative sample of breast cancer in our society and they provide a reliable insight of the cost of treatment.

Among the results, one that draws attention is the long interval of time between the first clinical and/or radiological alteration and the establishment of the diagnosis. We highlight that in this study, this step happened before patients were included into the program. A similar delay was also found by Rezende et al.^(9)^, who reported that patients with a clinical suspicion of breast cancer waited for a mean interval of 6.5 months for diagnostic confirmation in the public network. It is possible that many of these cases could have been diagnosed in a more agile manner, if there were a flow of scheduling in the private network (*via* PPP) for each case in which the biopsy could not be scheduled in the public network within one month. Of the patients in our study, approximately one third presented with locally advanced or metastatic disease at the time of diagnosis, similar to what was found by Trufelli et al.^(3)^, with the consequent negative implication on prognosis. Once the pathological diagnosis was obtained, the median interval to the first treatment in our study was considered excessive (Table 4) when compared to that observed in developed countries^(10,11)^. Among our patients, the main factor responsible for this delay was the very patient flow established by public-private partnership, which determined the return of patients with biopsy results to the UBS so that only then, as per norms established by the UBSs themselves, could they be directed to some oncologic treatment center. In developed countries, it is recommended that this interval be of less than 30 days, at the most^(8,11)^, due to the risk that greater intervals would allow the progression of the disease and worse prognosis. As an example of this recommendation, in a large retrospective study carried out with the North-American population by Bleicher et al.^(10)^, the median interval between the first consultation and surgical treatment was 29 days. Also, data from a study limited to North Carolina, in the United States, indicates a median interval between the biopsy and the first treatment of 22 days, documenting that delays longer than 60 days are harmful, especially for locally advanced or metastatic tumors at diagnosis^(12)^. Thus, in the case of future public-private partnerships, patients' return to the UBS should be eliminated, and the patient should have her treatment established or at least planned immediately at the very unit where the diagnostic biopsy was done.

As to the interval between the various phases of treatment, some findings may be compared to those described by Trufelli et al.^(3)^ in a public hospital in the state of São Paulo. In this publication, the median time of 55 days between the end of neo-adjuvant treatment and surgery was similar to ours, but the time delay to initiate adjuvant chemotherapy (48 days) after surgery was a lot longer. Despite the fact that there is no consensus as to the ideal time between termination of neo-adjuvant treatment and surgical treatment, we considered an interval longer than 30 days excessive (Table 4), due to the risk of progression of the disease between the maximal response to treatment and surgical intervention. In the specific case of neo-adjuvant treatment, the delays among our patients occurred due to the temporary loss of follow-up in two patients (for lack of comprehension as to the need to return in one case and serious social problems in another), besides brief limitations in surgical scheduling. The interval between surgery and the beginning of adjuvant treatment, as well as the interval until the beginning of radiation therapy, was considered adequate and consistent with what is recommended, which is a maximal interval of 3 and 4 months, respectively^(11,13)^ – except in four cases: three were due to delay of adjuvant treatment secondary to complications related to breast reconstruction surgery; one case was a result of clinical complications not related to cancer. It is worth pointing out the short time interval between the end of systemic treatment and the initiation of radiation therapy, which was better than what is recommended^(13)^; no data was found in Brazilian literature for comparison. Considering the relative lack of radiation therapy centers in the country ^(14,15)^ and the great discrepancy in regional availability, it is likely that the establishment of PPPs would allow a decrease in delays to initiation of radiation therapy.

Although the most important result of any PPP is the reduction in cancer mortality, the documentation of this result would require a large number of women treated in this program and would take several years to complete. The most immediate benefit that can be determined is the adoption of quality treatments in the shortest time possible. When compared to treatment at a public institution^(3)^, we achieved a shorterdelay until the beginning of adjuvant chemotherapy, and, following international recommendations^(13)^ a timely initiation of radiation therapy. It is possible that compliance with international treatment standards would be more plausible if the entire treatment could be done at the same center (whether public or private) in an integrated manner among the various specialties. This is the principle that determines the establishment of High Complexity Centers and Units in Oncology (CACONs and UNACONs) in the country, but their reach still does not cover all the demand. Therefore, in structuring a future PPP, the most important intervals to be monitored are clinical suspicion-mammogram-biopsy; biopsy-surgery; pathology report-adjuvant treatment.

From an economic point of view, since it contains a significant component of clinical assistance, the project was not considered by the Ministry of Health as adequate to fulfill the criteria of the “SUS Institutional Development Support Project” (as foreseen by Official Statement MS/GM No. 3,276)^(16)^. Therefore, the PRPO was fully financed by HIAE, after an agreement with the Municipal Government *Núcleo de Planejamento Estratégico da Secretaria Municipal de Saúde* (NUPES) and with the *Coordenadoria Regional Sul de Saúde*.

Even though no studies were found for comparison, using as reference the table of fees for procedures paid for by SUS to hospitals of the public sector^(17)^, it is probable that the value spent in diagnosis and treatment of the patients from our PRPO are higher than what is spent in the public healthcare network. As an example, using this table^(17)^ as a reference, SUS pays the public hospitals the value of US$ 54.16 for the a pathology report with hormone receptors status evaluation; US$ 391 for a mastectomy with lymphadenectomy; US$ 179 for a breast conserving surgery with lymphadenectomy; and US$ 240 for radiation therapy. The discrepancy among those values and our costs reflect, at least in part, the sub-standard values paid by the Ministry of Health to the service providers.

Oncology is among the most costly specialties in medicine, as a consequence of the high value of the diagnostic tests and innovative medications^(18)^. For a patient submitted to biopsy, breast conserving surgery, chemotherapy without trastuzumab (regime with taxane followed by anthracycline), radiation therapy, and 5 years of tamoxifen, our approximate cost reached US$ 25,500.00. It is possible to consider that this would be the cost of treatment (following international standards) for about 50% of the women diagnosed with breast cancer in Brazil (stages II and III, Her2 negative). If, on the other hand, adjuvant chemotherapy without the addition of a taxane (and without trastuzumab) were used, increasing by a few percentage points the risk of relapses (with an increase in metastatic cases and their consequent expenses), the median cost of treatment (identical to that described above, except for the chemotherapy) would be US$ 16,000.00. The addition of trastuzumab to the neoadjuvant or adjuvant treatment (during one year) would raise the cost to a total of US$ 95,000.00. Recently, trastuzumab was incorporated for use at SUS only in adjuvant or neo-adjuvant setting of breast cancer^(19)^, though the indication for use in metastatic disease may still be approved. Considering the values negotiated by the Ministry of Health^(19)^, one year with medication for a 60-kg patient would be approximately US$ 45,500.00, compared to the US$ 66,250.00 in our study (Table 3). Despite the substantial increase in the cost of the treatment, the addition of trastuzumab is considered cost-effective due to the decrease in risks of relapses and mortality conferred by the medication, resulting in lower subsequent expenditures^(20,21)^.

We found no data describing the cost of chemotherapeutical treatment for breast cancer in Brazil, whether in the public or private sector. However, once again, when the findings were compared to the values reimbursed by the Ministry of Health^(17)^, a large difference is noted. As an example, in SUS, reimbursement for chemotherapeutical treatment in the adjuvant setting for Her-2 negative breast tumors is around US$ 1,714.50 to US$ 2,400.00 per patient, depending on the pathological stage of the tumor^(17)^. We point out, however, that these values represent the amount reimbursed by SUS to hospitals that offer oncologic assistance, and may probably not reflect the total expenditure by the hospitals in the care of a given patient.

The data from our study do not discriminate the costs of treatment of metastatic disease, due to the small number of cases and the immense variation in costs, which depend on the clinical scenario of each patient, even though the costs were considered regarding the total amount spent on the project. In the United States, the mean cost per month of treatment for one patient with metastatic breast cancer is US$ 9,788.00, varying according to the use of systemic or oral chemotherapy, reaching a total value of US$ 250,000.00 per patient with a median survival of 2 years^(22)^. Data presented at the American Society of Clinical Oncology meeting in 2011 showed that 6 months of treatment with capecitabine, an oral chemotherapy agent, costs US$ 3 to 8 thousand in countries such as Argentina and Egypt, while that of an aromatase inhibitor, also used to treat metastatic disease, may cost a lot less (US$ 500.00 to US$ 1,400.00) ^(23)^. Within the context of budget destined to healthcare (US$ 45.5 billion in 2012)^(24)^ and more specifically to the treatment of cancer (US$ 1.2 billion in 2012)^(25)^, we observe how the treatment of breast cancer, in following international standards of excellence, could consume vast resources of this budget.

One limitation of our results is that there were expenses that could not be assessed, such as expenditures with human resources (pathologist, radiologist, radiotherapist, and oncologists), since these professionals were not hired for exclusive dedication to the project (as they were already members of the staff of HIAE and only dedicated a small part of their time to PRPO). Nevertheless, the study maintains its relevance, since it provides pioneer information on the main costs related to diagnosis and treatment of breast cancer in Brazil.

Based on what was observed at PRPO, a possible economic strategy would be that of limiting assistance strictly to what is offered by SUS, even if provided by private hospitals. For example, not offering trastuzumab to patients with metastatic Her-2 positive disease, not offering Aprepitant even in cases of highly emetogenic chemotherapy, avoiding placement of central venous catheters, and not proceeding with genetic tests, among others. A second strategy would be that of offering assistance in partnership only during the more critical phases of treatment and those most deficient at SUS, such as access to rapid diagnosis (making available core needle biopsies and mammotomiesat centers of excellence), and surgery within 30 days of the biopsy if the time schedule at SUS is not possible, besides radiation therapy. In systemic treatment, proportionally more expensive and with a greater availability at SUS^(15)^, this would be done within the structure of SUS. This would require an extremely rigorous reference and counter-reference flow chart so that all the benefit achieved through timely surgical treatment and radiation therapy is not lost in the interval of returning to SUS for systemic treatment.

## CONCLUSION

The expenses described provide relevant information about the costs associated with the diagnosis and treatment of patients with breast cancer, pointing to the need for cost reduction strategies in the private network in order to allow future large-scale public-private partnerships in oncology. Furthermore, the analysis of the flow we worked with enables the institution of priorities for future assistance in breast cancer, suggesting that patients' journeys to and from the private partner and the original public service should be avoided or optimized along the process of diagnosis and treatment, so that there are no delays with potential deleterious effects in the prognosis.
